# Development of ListeriaBase and comparative analysis of *Listeria monocytogenes*

**DOI:** 10.1186/s12864-015-1959-5

**Published:** 2015-10-06

**Authors:** Mui Fern Tan, Cheuk Chuen Siow, Avirup Dutta, Naresh VR Mutha, Wei Yee Wee, Hamed Heydari, Shi Yang Tan, Mia Yang Ang, Guat Jah Wong, Siew Woh Choo

**Affiliations:** Genome Informatics Research Laboratory, High Impact Research Building, University of Malaya, Kuala Lumpur, 50603 Malaysia; Department of Oral Biology and Biomedical Sciences, Faculty of Dentistry, University of Malaya, Kuala Lumpur, 50603 Malaysia; Genome Solutions Sdn Bhd, Suite 8, Innovation Incubator UM, Level 5, Research Management & Innovation Complex, University of Malaya, Kuala Lumpur, 50603 Malaysia; Computer Science and Engineering Department, University of Nebraska-Lincoln, Lincoln, NE 1468588-0115 USA

**Keywords:** ListeriaBase, *Listeria*, Comparative analysis

## Abstract

**Background:**

*Listeria* consists of both pathogenic and non-pathogenic species. Reports of similarities between the genomic content between some pathogenic and non-pathogenic species necessitates the investigation of these species at the genomic level to understand the evolution of virulence-associated genes. With *Listeria* genome data growing exponentially, comparative genomic analysis may give better insights into evolution, genetics and phylogeny of *Listeria* spp., leading to better management of the diseases caused by them.

**Description:**

With this motivation, we have developed ListeriaBase, a web *Listeria* genomic resource and analysis platform to facilitate comparative analysis of *Listeria* spp. ListeriaBase currently houses 850,402 protein-coding genes, 18,113 RNAs and 15,576 tRNAs from 285 genome sequences of different *Listeria* strains. An AJAX-based real time search system implemented in ListeriaBase facilitates searching of this huge genomic data. Our in-house designed comparative analysis tools such as Pairwise Genome Comparison (PGC) tool allowing comparison between two genomes, Pathogenomics Profiling Tool (PathoProT) for comparing the virulence genes, and ListeriaTree for phylogenic classification, were customized and incorporated in ListeriaBase facilitating comparative genomic analysis of *Listeria* spp. Interestingly, we identified a unique genomic feature in the *L. monocytogenes* genomes in our analysis. The Auto protein sequences of the serotype 4 and the non-serotype 4 strains of *L. monocytogenes* possessed unique sequence signatures that can differentiate the two groups. We propose that the *aut* gene may be a potential gene marker for differentiating the serotype 4 strains from other serotypes of *L. monocytogenes*.

**Conclusions:**

ListeriaBase is a useful resource and analysis platform that can facilitate comparative analysis of *Listeria* for the scientific communities. We have successfully demonstrated some key utilities of ListeriaBase. The knowledge that we obtained in the analyses of *L. monocytogenes* may be important for functional works of this human pathogen in future. ListeriaBase is currently available at http://listeria.um.edu.my.

**Electronic supplementary material:**

The online version of this article (doi:10.1186/s12864-015-1959-5) contains supplementary material, which is available to authorized users.

## Background

The *Listeria* genus consists of facultative anaerobic, Gram-positive, flagellated rods ubiquitously distributed in the environment. Some of the known species of this genus are *L. monocytogenes*, *L. ivanovii*, *L. marthii*, *L. innocua*, *L. welshimeri*, *L. seeligeri*, *L. grayi*, *L. rocourtiae*, *L. fleischmannii* and *L. weihenstephanensis*. Of these known *Listeria* species, *L. monocytogenes* and *L. ivanovii* are the most significant pathogens [1, 2]. *L. monocytogenes* affects both animals and humans (infant, elderly, pregnant women and immunocompromised, a risk group commonly referred to as YOPIs) and causes listeriosis, a severe foodborne disease that causes infections particularly on the central nervous system like meningitis, meningoencephalitis, brain abscess and cerebritis [3–7]. There is also the non-invasive form of listeriosis caused by *L. monocytogenes* in healthy people leading to outbreak, as the individuals developed febrile gastroenteritis [8, 9]. It has also been reported that *L. ivanovii* can cause infections mainly in ruminants, typically causing septicemic disease, neonatal sepsis and abortion [3–7]. *L. ivanovii*-caused infection in human are rare and only seven cases have been reported since 1955 [2]. Interestingly, the two *Listeria* pathogens are genetically closely related to some of the non-pathogenic *Listeria* spp. For instance, *L. monocytogenes* is akin to *L. innocua* and *L. marthii* [10], whereas *L. ivanovii* is akin to *L. seeligeri* [11, 12]. Some previous evidence indicate that a common pathogenic ancestor containing the key virulence genes diverged to give rise to the modern pathogenic and non-pathogenic *Listeria* species and strains about 47 million years ago [13]. For instance, gene loss events, including loss of virulence-associated genes such as the *prfA* cluster during the evolution of *Listeria*, have played a critical role in the transition of *Listeria* species from facultative pathogen to saprotroph, suggesting that *Listeria* has a tendency to evolve through loss of virulence rather than acquisition of virulence characteristics. Surprisingly, a number of non-pathogenic isolates still carry some of the virulence genes [13].

Due to the pathogenicity of *L. monocytogenes* and its capability to thrive in harsh environments, previous genome sequencing and research efforts were largely focused on this species [2, 14–21]. Several genomic databases have been developed to allow researchers to investigate the different aspects of *L. monocytogenes*. One of these databases is the *Listeria monocytogenes* Database (http://www.broadinstitute.org/annotation/genome/listeria_group), which was developed and maintained by the research group of Broad Institute. This database facilitates comparison across different *L. monocytogenes* genomes, for example, through the dot-plot analysis. Another existing database, Proteome Database LEGER [22] supports functional genome studies of *L. monocytogenes* and its non-pathogenic relative, *L. innocua*. ListiList (now integrated in GenoList multi-genome browser [23]) was also introduced to provide a platform for the analysis of *L. monocytogenes* and *L. innocua*, with the addition of *L. welshimeri* in GenoList. PATRIC [24] provides genomic and virulence factors information of some of the *Listeria* strains, however, lacks the functionalities for comparative pathogenomic analysis of *Listeria* strains by comparing, clustering and visualizing their virulence gene profiles.

With the advances in next-generation sequencing technologies, many genomes of *Listeria* spp. have recently been sequenced by researchers [2, 14]. With the increasing number of *Listeria* genomes, comparative analysis of these genomes will help to study the different aspects of *Listeria* spp. including its evolution, diversity, genetics, biology and pathogenicity. More importantly, this powerful approach allows the study of pathogen evolution of *Listeria* spp., for example, by examining the genetic or genomic differences between the non-pathogenic and pathogenic *Listeria* strains/genomes. It is crucial to understand the evolution of genes expressing virulence factors, which may also help in the development of genetic and genomic criteria for pathogenic strains, including the development of assays for the detection of pathogenic *Listeria* strains [13, 25]. Moreover, any new knowledge generated from these analyses may lead to better understanding of *Listeria* pathogenicity which could be important for the diagnosis and management of the *Listeria*-caused diseases and drug design. To facilitate *Listeria* research, a specialized and centralized genomic resource and analysis platform for *Listeria* is critical, for the storage of the vast amount of genome sequences and genomic information, and for analytical purposes, particularly in the field of comparative genomics. With that in mind, we constructed a freely available online platform, ListeriaBase, hosting useful genomic data and annotations of *Listeria* species, regardless of whether they are pathogenic or non-pathogenic. Most importantly, in addition to its intuitive web interfaces, ListeriaBase is also an analysis platform, where the users need not go elsewhere, but can perform some of the important comparative analyses using our in-house designed comparative analysis pipelines. Using the popular scripting languages like Python, Perl, BioPerl [26] and R, we developed the PGC tool for comparing genomes, PathoProT for comparative pathogenomics analysis of the *Listeria* genomes and ListeriaTree for phylogenetic classification of the *Listeria* strains. Apart from these we have also incorporated the BLAST search tool for the homology search, real-time search feature for searching keywords within the ListeriaBase and an AJAX-based genome browser for visualizing the *Listeria* genomes in the ListeriaBase. All these analytical tools and the features were designed with the idea of making ListeriaBase a resourceful, comprehensive and user-friendly platform dedicated to *Listeria* research, where the researchers can retrieve their desired data and process them to generate useful findings that may have a deep impact on better understanding of the biology, evolution, diversity, and virulence of *Listeria*.

## Construction and content

### Data collection and preprocessing

ListeriaBase currently hosts 285 genome sequences covering 10 *Listeria* species (*L. grayi*, *L. innocua*, *L. ivanovii*, *L. marthii*, *L. monocytogenes*, *L. seeligeri*, *L. welshimeri*, *L. fleischmannii*, *L. weihenstephanensis* and *L. rocourtiae*) (Table 1) that were obtained from National Center for Biotechnology Information (NCBI) [27]. Of the 285 genome sequences, 49 are complete genomes (chromosome data) and 236 are draft or incomplete genomes (either contigs or scaffolds data). To ensure the uniformity in the annotations of these genomes which is important for comparative analysis, all genome sequences were annotated by uploading their sequence files to Rapid Annotation using Subsystems Technology (RAST) [28], a fully automated server that provides identification of protein encoding region and gene functions. The RAST-predicted protein sequences and other annotations (e.g. gene functions, amino acid length, protein hydrophobicity, molecular weight, etc.) were downloaded for downstream analyses such as protein subcellular localization which gives important clues to identify potential key drug targets in an organism. Bacterial subcellular localization of *Listeria* strains were analyzed using PSORTb version 3.0, which is a well-established software for the prediction of the subcellular localization of proteins for prokaryotes [29]. The predicted subcellular localization information for each RAST-predicted protein was stored in MySQL tables of ListeriaBase. In general, the subcellular localization of *Listeria* proteins were predicted and classified into five groups: cytoplasmic, cytoplasmic membrane, extracellular, cell wall and unknown.

**Table 1:** List of *Listeria* species and the number of genomes in ListeriaBase (as on 29^th^ August 2014)

**#**	Species	# Draft genomes	# Complete genomes
1	*L. grayi*	2	0
2	*L. innocua*	3	1
3	*L. ivanovii*	1	2
4	*L. marthii*	1	0
5	*L. monocytogenes*	223	44
6	*L. seeligeri*	2	1
7	*L. welshimeri*	0	1
8	*L. fleischmannii*	2	0
9	*L. weihenstephanensis*	1	0
10	*L. rocourtiae*	1	0

### ListeriaBase implementation

ListeriaBase was designed based on 4-tier web application architecture: client workstation, web server, application server and database server, implemented using LAMP solution stack (software bundle for Linux, Apache, MySQL and PHP). The ListeriaBase website was built using PHP and followed the MVC (model-view-controller) framework to separate logic, presentation and application data into three interconnected parts. Client-side scripting was done with jQuery, a feature-rich JavaScript library. jQuery enhances user interaction with the web pages through the use of AJAX (Asynchronous JavaScript and XML) communication libraries for asynchronously transferring data between the client workstations and server-side programs. Apache web server handles requests from web clients and communicates with the back-end servers to execute the requests. Server-side operations are performed in a Linux server (CentOS 5.8) through in-house scripts (Perl, Python and R). MySQL database is responsible for storing annotated sequence data.

### The Graphical User Interface (GUI)

#### Overview of ListeriaBase

**Fig. 1:**
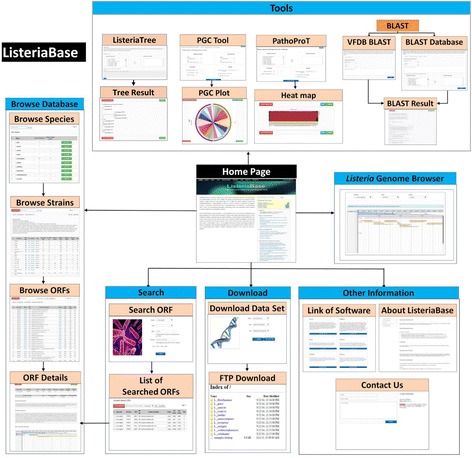
Overview of ListeriaBase architecture

The ListeriaBase homepage features a brief description of the genus *Listeria* in the main panel along with manually compiled information that are related to *Listeria* such as news & conferences, blogs & other information and the most recent published papers in the side panel. Users can browse, search and access *Listeria* genome sequences and annotation data through the provided user-friendly web interfaces. For instance, the ‘Browse’ feature allows users to browse the annotations through the detail links provided at the right side of each populated list: (i) list of available species in ListeriaBase and the number of draft or complete genomes for each species; (ii) brief description about the species, list of strains and their properties (genome size, GC content, number of contigs, CDSs, tRNAs and rRNAs, along with the links to their taxonomic and assembly details); (iii) list of strain-specific open reading frames (ORFs) and their details (ORF ID, ORF type, functional classification, contig ID, start position and stop position); and (iv) ORF-specific information (subcellular localization, hydrophobicity, molecular weight, amino acid sequences and nucleotide sequences). Furthermore, a real-time data search feature was implemented for fast and smooth searching of the queries in the ListeriaBase. The database also provides options to visualize the genomes and analyze the genomic features using the built-in genome browser. In addition, ListeriaBase is equipped with a number of analytical tools such as sequence similarity search tools (variants of BLAST), in-house designed tools such as PGC, PathoProT and ListeriaTree (Fig. 1).

## Utility

### Browsing ListeriaBase

Users can browse the Listeria species and genomes using the “Browse” tab in the homepage of ListeriaBase. All *Listeria* species and genomes currently available in ListeriaBase are displayed in a tabular fashion. For each species, the number of draft and complete genomes available are shown along with a “View Strains” button. By clicking on the “View Strains” button, it will lead the users to the “Browse Strains” page showing all available strains of that species and their general annotations such as genome size (Mbp), GC Content (%), number of contigs, ORFs, tRNAs and rRNAs. Each strain is also linked to external resources such as their corresponding taxonomic classification and assembly page in NCBI through the “Taxon” and the “Assembly” links respectively and also the details of their Multi Locus Sequence Typing (MLST) data in Institute Pasteur MLST Databases (www.pasteur.fr/mlst). Besides that, by clicking on the “Details” button of a *Listeria* strain, it will open the “Browse ORF” page, containing all ORFs/genes in the genome of the strain and annotations such as predicted function start and stop positions in a tabular form. If users want to access the original and other relevant information of an ORF such as locus_tags in GenBank, they can click on the ORF ID of interest to open the corresponding page of this ORF/gene in the GenBank. Furthermore, users can click on the details button of an ORF if they want to open the “ORF Detail” page showing the detailed information of the ORF such as gene type, start and stop positions, lengths of nucleotide and amino acid sequences, the ORF sequences (both nucleotide and protein), functional classification, strand, subcellular localization, hydrophobicity (pH) and molecular weight (Da), number of transmembrane helices and its isoelectric point (pI). We have also incorporated the JBrowse [30, 31] in the “ORF Details” page to allow users visualizing the location of the ORF in the genome along with its relevant details. The users can download the ORF details through the provided “Download” button.

### Real-time keyword and text-based searches

ListeriaBase hosts a huge amount of *Listeria* genomic data and annotation. With the advent of high-throughput sequencing technologies, these data are expected to considerably increase as more genomes are sequenced in the future. Therefore, an intuitive web-based GUI allowing users to rapidly search a large volume of genomic data in real time is vital.

In ListeriaBase we implemented a real-time search engine to facilitate seamless search capability, complementing the ‘Browse’ feature by allowing users to query annotations rapidly and in real-time through the use of AJAX technology. This AJAX technology, which is a combination of different technologies such as HTML, CSS, DOM, XML and JavaScript, allows our database to have a wider variety of controls and functions. This results in the reduction of workload on server considerably, allowing heavy analyses to be processed simultaneously. The design of the search function significantly speeds up the searching process in a large dataset. The users can streamline their search by using the search parameters such as species, strain, ORF ID, keywords of functional classification and type of sequence; the system will retrieve the matches in real-time as soon as users input the desired keywords or even suggest the probable keywords to speed up the querying process.

### Sequence searches

The built-in BLAST [32] in ListeriaBase allows users to search or compare a query sequence against the database. Through this tool, users can perform nucleotide comparisons (BLASTN), whole genome nucleotide comparisons (BLAST Whole Genome), protein comparisons (BLASTP), and nucleotide with protein comparisons (BLASTX). Users have the options to select whether the search will be against (i) all *Listeria* genomes, (ii) a single or multiple genomes, or (iii) in the case of nucleotide search, against genomic sequences or protein-coding sequences only and can set the cut-off for BLAST expect value and turn on/off a filter for low compositional complexity regions. Since virulence factors provide important clue for bacteria pathogenicity, we designed and incorporated VFDB BLAST specifically for searching the Virulence Factor Database (VFDB) [33] into ListeriaBase. Using the VFDB BLAST tool, users can examine whether their sequences are virulence genes based on sequence similarity. Options for VFDB BLAST include BLASTN (for nucleotide sequences) and BLASTP (for protein sequences) programs, with similar parameters to that of the standard BLAST.

### Interactive *Listeria* genome browser

To give users a seamless browsing experience, we incorporated AJAX-based JBrowse into ListeriaBase [30, 31]. Using this genome browser, users can visually navigate *Listeria* genome sequence and annotation data on the fly. Unlike traditional genome browsers such as GBrowse [34], JBrowse supports fast and smooth animated genome navigation, offering seamless interaction for the users while using the genome browser. Furthermore, JBrowse enables high speed visualization of contigs, DNA sequences, RNA sequences and genome annotation results based on the parameters set by users such as *Listeria* species, strains and contig number. The users can also click on the ORF in the Genome Browser to view all the relevant information of the ORF such as its name, type, description, position, length as well as its other attributes like its GC (%), ORD ID, Sequence ID, subcellular localization, number of transmembrane helix and its isoelectric point (pI), along with the nucleotide sequence in the fasta format.

### Data download

Users can download all the genome sequences and annotations available in ListeriaBase through the ‘Download’ page. Through the provided interactive GUI forms, users can select which data and annotations to download. Alternatively, users can download these data and annotations with a File Transfer Protocol (FTP) download option provided in the ‘Download’ page.

### In-house designed bioinformatics tools

#### ListeriaTree- constructing *Listeria* phylogenetic tree

As mentioned above, many evidence have indicated that the modern day pathogenic and non-pathogenic *Listeria* species have diverged from a common pathogenic ancestor containing the key virulence genes through the events of gene loss about 47 million years ago [13]. Phylogenetic study of the *Listeria* has thus become an important aspect in order to understand the evolutionary relationships between different species. This prompted us to develop ListeriaTree, a tool for the phylogenetic classification of the *Listeria* stains. ListeriaTree is an automated pipeline written in Perl that was incorporated into the ListeriaBase. Using the tool, users can generate phylogenetic trees of their *Listeria* strains of interest based on genes such as 16S rRNA gene, *gyrB*, *groEL*, *sigB*, or *actA*. Previous studies showed that 16S rRNA sequence analysis is an accurate and rapid method for identifying most unknown bacteria to the genus level, whereas the other genes mentioned above may be more effective at the species level [35–43]. However, it must be noted that *actA* cannot be chosen to create phylogenetic trees for non-pathogenic strains.

To use the ListeriaTree, users only needs to follow two steps: (i) select marker gene used for the construction of a phylogenetic tree, and (ii) choose a list of strains in ListeriaBase to be included in the tree through our online web form. Users can also choose the option to submit their sequence of interest (in FASTA format) along with the selected sequences from the ListeriaBase for generating the phylogenetic tree.

Once users submit their jobs, ListeriaTree pipeline starts retrieving all sequences of the marker genes of the user-selected strains (as well as the user-submitted sequence if it is applicable) and store them into a temporary FASTA file for alignment using MAFFT (Multiple Alignment using Fast Fourier Transform) [44]. ListeriaTree pipeline will call FastTree program [45] to construct a phylogenetic tree using the MAFFT-generated multiple alignment file. FastTree will construct the phylogenetic tree in five stages, which includes Heuristic neighbor-joining in the first stage to get a rough topology. In the next stage it attempts to reduce the length of the tree by using a combination of nearest-neighbor interchanges (NNIs) and subtree-prune-regraft moves (SPRs). The software will further improve the topology and the branch lengths of the tree by using maximum-likelihood rearrangements. In the final stage, FastTree will quickly estimate the reliability of each split in the tree. By default, FastTree computes local support values by resampling the site likelihoods 1000 times and using the Shimodaira-Hasegawa test on the three alternate topologies (NNIs) around that split. FastTree outputs the phylogenetic tree in a Newick format, and then converts it into the SVG format using the Newick Utilities for visualization [46]. ListeriaTree will display the final image of phylogenetic tree for visualization in web browser.

### PGC- an automated pipeline for pairwise genome comparison and visualization

In many cases, researchers may be interested in studying the genetic differences among the *Listeria* genomes. Therefore, we integrated an in-house developed Pairwise Genome Comparison (PGC) tool for comparing two user-selected genome sequences. Researchers have options to select two *Listeria* genomes of interest from the ListeriaBase or to upload their own *Listeria* genome sequence and compare with the strain/genome in the ListeriaBase through our custom GUI form.

PGC aligns the user-selected genomes using the NUCmer algorithm [47]. Once the genomes are aligned, PGC will parse the results to Circos [48] for generating a circular ideogram layout and to show the relationship between pairs of positions, with karyotypes and links encoding the position, size and orientation of the related genomic elements. The circular ideogram will give users a better insight into the genetic variation such as deletions, insertions and translocations between the two user-selected genome sequences, providing a clear representation of the genome structure of these strains. Users can download NUCmer genome alignments and the generated Circos plot using the ‘Download’ button in the PGC result page or opt for the analysis results to be directly sent to them through emails.

The multi-step process of the PGC pipeline was automated using our in-house Perl scripts and users can usually get the results within a few minutes. For flexibility in the analysis, users can set three parameters in PGC, based on their preferences or research needs prior to the submission of their analysis jobs to our server through a GUI: (i) Minimum Percent Genome Identity (MPGI); (ii) Link threshold (LT) which removes the links according to user-defined value; and (iii) Merge Threshold (MT) that allows merging of links based on user-defined value. By default, the thresholds of MPGI, LT and MT are set to be 95 %, 1 kbp and 0 base pairs respectively.

### PathoProT- an automated pipeline for comparative virulence gene analysis

As bacterial pathogenicity is a major concern for the public, we have customized and incorporated in ListeriaBase, our in-house designed Pathogenomics Profiling Tool (PathoProT), allowing users to identify the putative virulence genes and compare the virulence profiles across different *Listeria* strains [49]. Virulence factors can be grouped into distinct categories, e.g., bacterial toxins, hydrolytic enzymes and cell surface proteins attachment [50]. Most of the virulence factors are toxins which can be classified as either endotoxin or exotoxin [19, 20]. The PathoProT pipeline was developed and automated using in-house Perl and R scripts. PathoProT first predicts the virulence genes in the *Listeria* genomes that are selected by users through our provided GUI form. For each genome, the PathoProT will predict virulence genes by performing a BLAST search [51–54] of the RAST-predicted proteins against the experimentally verified virulence genes in the VFDB database (version 2012 containing a total of 19,775 proteins). The putative virulence genes will be identified based on the user-defined cutoff. The default parameters of the BLAST search are set at 50 % sequence identity and 50 % sequence completeness, but users can alter these parameters based on their desired stringency level. The automated PathoProT pipeline will organize the information about the strains and identified virulence genes into a data matrix format and then hierarchically cluster these virulence genes and strains for visualization in a heat map using R scripts. Through this heat map, users can answer many interesting biological questions such as the putative virulence genes identified in each strain, the differences between non-pathogenic and pathogenic strains, and the strains having similar virulence gene profiles.

## Discussion

The pathogenicity of *L. monocytogenes* and its ability to thrive in harsh environment has made it an important topic of study for years. Considering its importance and as a well-studied *Listeria* species, here we used *L. monocytogenes* as a case study to demonstrate the utilities of ListeriaBase and its tools.

### Genomic features of *L. monocytogenes*

We examined the genomic features of 44 complete genomes of *L. monocytogenes*. It should be noted that only the complete genomes were used in our analyses in order to have a more accurate and high quality results in our analyses. The 44 strains spanned all *Listeria* lineages except for lineage IV which currently has only one draft genome (Table 2). These strains were isolated from different geographical locations including USA, UK, France, China and Germany.

**Table 2:** Summary of the 44 *L. monocytogenes* genome annotations

Lineage	Strain	Serotype	Size (bp)	# ORFs	# tRNAs	GC (%)	Isolated from	Country	Year of isolation
Lineage I	07PF0776	4b	2,901,562	2942	67	38.04	Human myocardial abscess	USA	-
	ATCC 19117	4d	2,951,805	2957	67	37.99	Sheep	USA	-
	CLIP 80459	4b	2,912,690	2915	67	38.06	Clinical outbreak of listeriosis	France	-
	L312	4b	2,912,346	3045	67	38.06	Cheese	-	-
	F2365	4b	2,905,187	2920	67	38.04	Cheese	USA	1985
	LL195	4b	2,936,689	2920	67	38.01	-	Switzerland	1983 – 1987
	SLCC2482	7	2,972,810	2968	67	37.95	Human	-	1966
	SLCC2378	4e	2,972,172	2968	66	37.95	Poultry	-	-
	SLCC2540	3b	2,966,146	2994	67	38.08	Human	USA	1956
	SLCC2755	1/2b	2,907,142	2972	67	38.01	Chinchilla	-	1967
	J1816	4b	2,947,460	3060	58	37.97	Turkey deli meat	USA	2002
	J1-220	4b	3,032,271	3088	67	37.94	Vegetable	USA	1979
	CFSAN006122	-	2,906,670	2922	67	38	Cheese	USA	2013
	J2-064	1/2b	2,943,218	2945	58	38	Cow	-	-
	NE dc2014	-	2,904,662	2920	67	38	Cheese	-	-
	J2-1091	-	2,981,886	3025	67	38	Animal	USA	1995
	J1776	4b	2,953,719	2995	67	37.9	Turkey deli	USA	2002
	J1817	4b	2,953,716	2999	67	37.9	Turkey deli	USA	2002
	J1926	4b	2,953,708	2996	67	37.9	Turkey deli	USA	2002
	N1-011A	-	3,094,342	3169	67	38	-	-	-
	R2-502	1/2b	3,034,043	3079	67	37.9	-	-	1994
	WSLC1042	4b	2,942,168	2974	67	38	-	Germany	-
Lineage II	08-5578	1/2a	3,032,288	3112	58	37.96	Human blood specimen	Canada	2008
	08-5923	1/2a	2,999,054	3063	58	37.96	Human	Canada	2008
	10403S	1/2a	2,903,106	2944	67	38.03	Human skin lesion	USA	1968
	EGD-e	1/2a	2,944,528	2996	67	37.98	Rabbit	UK	1926
	Finland 1998	3a	2,874,431	2904	67	38.05	-	Finland	1998
	FSL R2-561	1/2c	2,973,801	3051	67	37.96	-	-	-
	J0161	1/2a	3,000,464	3060	58	37.86	Human listeriosis outbreak	-	-
	SLCC2372	1/2c	2,840,185	3037	67	38.26	Human	UK	1935
	SLCC2479	3c	2,976,958	3031	65	37.93	-	-	1966
	SLCC5850	1/2a	2,882,234	2976	67	38.04	Rabbit	UK	1924
	SLCC7179	3a	2,972,254	2927	67	37.95	Cheese	Austria	1986
	NCCP No. 15743	1/2a	2,803,433	2868	67	38.1	-	-	-
	6179	1/2a	3,010,620	3071	49	37.9	Cheese	-	-
	C1-387	1/2a	2,988,947	3043	67	38	Turkey breast	New York	1999
	EGD	1/2a	2,907,193	2969	67	38	Animal	-	1926
	J2-031	1/2a	2,958,908	3024	67	38	Cow	-	1996
	R479a	1/2a	2,944,998	3008	58	37.9	Smoked Salmon	-	-
	WSLC1001	1/2a	2,951,235	3031	67	38	-	Germany	-
Lineage III	HCC23	4a	2,976,212	3048	67	38.19	Catfish brain	USA	-
	L99	4a	2,979,198	2911	67	38.19	Cheese	Netherlands	1950
	M7	4a	2,976,163	3049	67	38.19	Cow’s milk	China	-
	SLCC2376	4c	2,941,360	2839	67	37.99	Poultry	-	-

(All the genomes referred in the table are complete genomes)

**Fig. 2:**
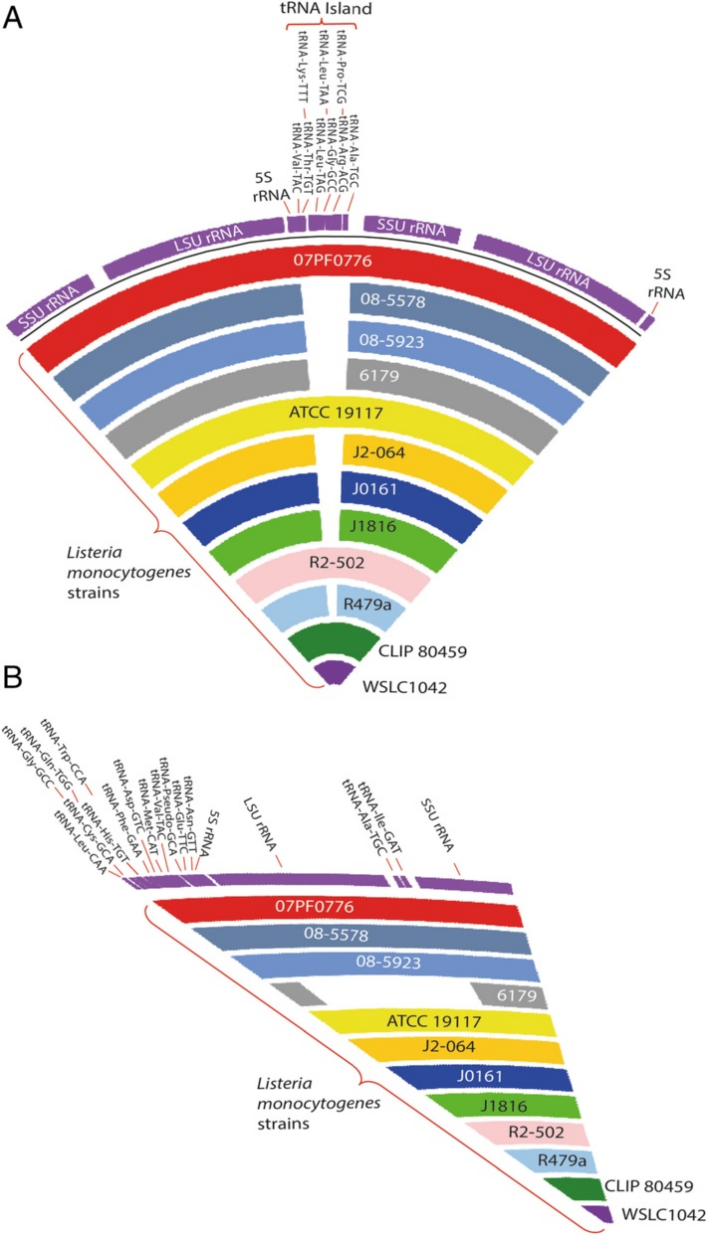
Genome comparison and visualization of multiple *L. monocytogenes* strains. **a** A tRNA island (TI1) containing 9 tRNA genes was located between two rRNA operons absent in the genomes of J1816, J0161, 08–5578, 08–5923, R479a, 6179 and FSL J2-064 causing lower number of tRNAs observed in these strains compared to other *L. monocytogenes* strains. **b** tRNA Island 3 (TI3) was absent in the complete genome of *L. monocytogenes* 6179*,* but present the rest of the strains

The number of functional genes ranged from 2839 to 3169. The average number of tRNA genes of these *L. monocytogenes* strains was approximately 67, but some strains (J1816, J0161, 08–5578, 08–5923, R479a and FSL J2-064) have lower number of tRNA genes, e.g., 58 tRNA genes despite being complete genomes. Those strains have 9 tRNA genes fewer as compared with others (Table 2 and Fig. 2), due to the absence of tRNA Island 1 (TI1) located between 2 of the rRNAs in the genome of *L. monocytogenes* SLCC5850 (Additional file 1: Table S1 and Additional file 2: Figure S1). More strikingly, *L. monocytogenes* 6179 has the lowest number of tRNA genes (49 tRNAs) due to the absence of two tRNA genomic islands (TI1 and TI3) (Additional file 1: Table S1, Fig. 2 and Additional file 2: Figure S1).

### Phylogenetic analysis of *Listeria monocytogenes*

To test out the in-house developed pipeline of ListeriaTree, we first constructed two phylogenetic trees based on the 16S rRNA gene sequences of the representative *Listeria* strains along with the representative strains of other genus such as *Campylobacter*, *Escherichia*, *Salmonella*, *Shigella*, *Yersinia* and *Vibrio*. The first tree was generated using the well-established tool MEGA (Additional file 3: Figure S2A) and the other using our in-house developed ListeriaTree tool (Additional file 3: Figure S2B). The classification in the ListeriaTree-generated tree was generally consistent with the classification in the tree generated from the well-established tool MEGA.

To examine whether 16S rRNA gene can discriminate *L. monocytogenes* strains into their respective lineages, a 16S-based tree was reconstructed with all 44 *L. monocytogenes* strains using ListeriaTree. As reported in previous studies [55], our results also showed that the 16S rRNA gene failed to discriminate the three lineages (Additional file 4: Figure S3A). But when we reconstructed the phylogenetic trees using the other 4 genes individually provided in ListeriaTree, in each of the four trees they were clearly clustered into their respective lineages (Additional file 4: Figure S3B-E). These results indicated that 16S rRNA genes might be effective in classifying the strains at the genus level, whereas the 4 genes were found to be more effective in differentiating the strains into their respective lineages. Here we have demonstrated that ListeriaTree pipeline can be used for classification of *Listeria* strains using the provided genes.

### Comparative genomic analysis

**Fig. 3:**
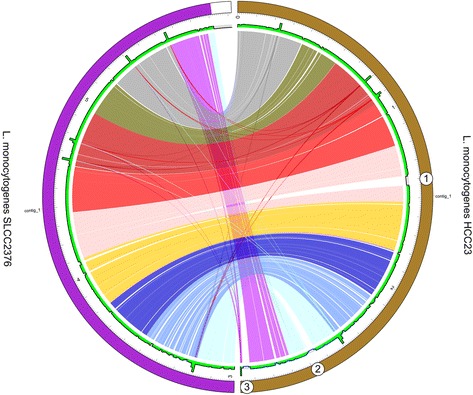
Pairwise genome comparison between the *L. monocytogenes* SLCC2376 and *L. monocytogenes* HCC23 from the lineage III. Three noticeable gaps and insertions can be observed and labelled as 1, 2 and 3 in circles found in the genome sequences of HCC23 and SLCC2376, which predicted to be putative prophage regions by PHAST. Two are intact prophages, whereas another one is a questionable (close to complete) prophage. The green track indicates the histogram bars. Each 10 Kbp window in the diagram is assigned by a histogram bar. The height of each bar illustrates the total number of bases of the opposite genome aligned to this 10 Kbp window region. The upper border of the grey area delineates 10 Kbp height. If the height is higher than the 10 Kbp, it may indicate the genomic region is not specific or containing repetitive regions. A gap may indicate unmapped region which could be an insertion e.g. prophages

When examining the genomic features of the lineage III strains, we found the genome size of SLCC2376 (2.94 Mbp) was generally smaller than the rest of the three strains in the same lineage (approximately 2.97 Mbp). This prompted us to further investigate the differences between the genome of SLCC2376 with the genomes of other lineage III strains using the PGC tool provided in ListeriaBase. Interestingly, the comparison between the genomes of SLCC2376 and HCC23 revealed not only significant rearrangement events, but also 3 noticeable insertions (or gaps in the genome of SLCC2376) in the genome of HCC23 as shown in the PGC plot (Fig. 3). The three noticeable insertions were also clearly observed in the rest of the two lineage III strains, albeit without rearrangements, when we compared SLCC2376 with L99 and M7 (Additional file 5: Figure S4). Further examinations of the three inserted genomic regions of HCC23, L99 and M7 in the *Listeria* genome browser of ListeriaBase revealed the presence of phage-related genes such as phage integrase, phage capsid protein, tail tape-measure protein, holing, putative tail or base-plate protein, phage portal (connector) protein and phage terminase.

We wondered whether these inserted genomic regions were horizontally transferred prophages. To examine this, we tried to predict the presence of prophages in the genomes of SLCC2376, HCC23, L99 and M7 using the online PHAge Search Tool (PHAST) [56]. PHAST identified three distinct prophage regions in the genome of HCC23. The first two were predicted as intact prophages indicating that these prophages were recently acquired by HCC23, whereas the third one was a questionable (close to complete) prophage (Additional file 6: Figure S5). PHAST also identified the same prophage regions in both genomes of L99 and M7. The genomic positions of the three prophages fitted very well to the positions of the insertions that we previously observed in these strains, suggesting that these prophages were horizontally transferred into the genomes of HCC23, L99 and M7, but not the SLCC2376 genome. No prophages were predicted in the genome of SLCC2376. Therefore, one of the reasons for the smaller genome size of SLCC2376 could be due to the absence of the three prophages that we observed in the other three lineage III strains. This demonstrates how PGC tool of the ListeriaBase can be very useful and be used to identify and visualize the genetic differences between different *Listeria* genomes.

### Pan-genome analysis

While analyzing the genome composition of the different lineages of *L. monocytogenes* in the ListeriaBase, we observed the presence of both type II restriction modification (RM II) system and the clustered regularly interspaced short palindromic repeat (CRISPR)-associated proteins (Cas) defense system in all lineage III strains, except the SLCC2376 which has only the RM II but not the CRISPR-Cas defense system (Additional file 7: Table S2). Many strains of lineages I and II did not have these systems. RM II is the most prevalent and simplest among the restriction modification systems because their restriction and modification enzymes work separately and only require Mg^2+^ as cofactor [14]. The RM II system of the lineage III strains consist of both restriction enzyme NgoPII (EC 3.1.21.4) and DNA-cytosine methyltransferase (EC 2.1.1.37), that can recognize the specific sequences of foreign DNA and degrade them into pieces [57]. Interestingly, we found the restriction enzyme NgoPII (EC 3.1.21.4) was absent in all lineage I and II strains, suggesting that the enzyme is specific to lineage III strains.

Furthermore, the adaptive immunity system CRISPR-Cas also acts as a defensive mechanism by recognizing and cleaving invading genetic elements [58]. We found the presence of the complete CRISPR-Cas system in all lineage III strains (except SLCC2376), but this system was absent in majority of the strains from the lineage I and II. Taken all together, the presence of the complete Type II restriction modification system in all lineage III strains and the CRISPR-Cas in majority (if not all) of these strains might help this lineage to protect themselves from the invasion of foreign DNA such as phages. Based on this, we hypothesized that the lineage III strains may generally have a closed pan-genome or conserved genome structures compared to other lineages due to the presence of these defense systems [57–60].

To test our hypothesis, we performed pan-genome (as well as core genome) analysis using the PGAP analysis pipeline [61] for *L. monocytogenes* lineages I, II and III by extrapolating the complete genome data of each lineage. To predict the pan-genome and core genome sizes of *L. monocytogenes*, we used N genomes to calculate gene clusters and core clusters, where N is the number of *L. monocytogenes* genomes (N = 1,2,3…43,44). The pan-genome size and core genome for each of the permutations of genome comparisons was predicted for each N genome. The curve for the pan-genome size can be represented by the following mathematical function of Y = 2735.2287 X^0.5^ + 544.4458 (R^2^ = 0.99) where, Y represents pan-genome size, while X represents number of sequenced genomes (Pan-genome size = infinite when X → ∞). Positive value for exponent of X indicates an open pan-genome whereas a negative value indicates a closed pan-genome, meaning no new gene to be found when a new genome is sequenced.

**Fig. 4:**
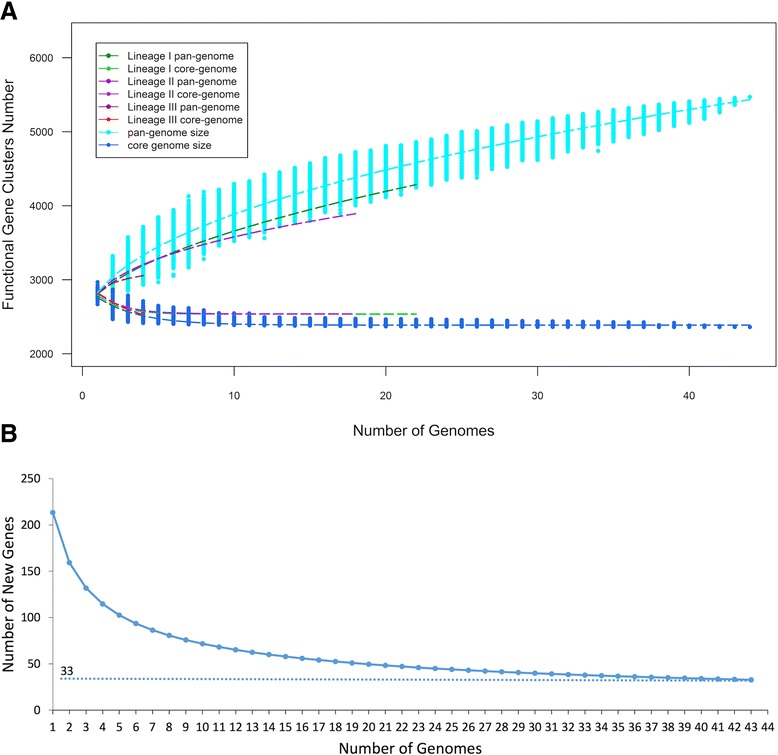
Pan-genome and core genome of *L. monocytogenes* size prediction. **a** The extrapolation of pan-genome and core genome sizes leads to two separate leaves: the upper leaf represents the pan-genome size and the bottom leaf represents the core genome size. **b** Curve for the number of expected new genes detected on the subsequent addition of *L. monocytogenes* genomes. 33 new genes predicted to occur for each addition

We predicted the size of pan-genome and core genome for each of the permutations on the 44 genomes selected in this study using the protein sequences available at ListeriaBase. As anticipated, our data showed the lineages I and II strains have open pan-genomes. In contrast, the lineage III (currently with only 4 available complete genomes) showed a closed pan-genome, reflecting that this lineage might have a conserved genome structure compared to the other lineages (Table 3 and Fig. 4a).

**Table 3:** Mathematical function for determining pan-genome of the lineages

Lineage	Formula	Pan-genome
I, II, III	Y = 585.7852 X^0.448^ + 2242.6997	Open
I	Y = 353.6843 X^0.532^ + 2453.6727	Open
II	Y = 584.9790 X^0.3580^ + 2245.4068	Open
III	Y = −538.9837 X^−0.402^ + 3364.3139	Closed

Y represents the pan-genome size while X represents the number of sequenced genomes (Pan-genome size = infinite when X → ∞). The negative value of exponent for X as shown in the formula indicates that lineage III has a closed pan-genome, meaning no new gene to be found when a new genome is sequenced

The 44 complete genomes of *L. monocytogenes* under the present study showed a pan-genome size of 5469 gene clusters that comprised of 2360 (43.1 %) core gene clusters (shared by all strains) and 3109 (56.8 %) accessory gene clusters. Accessory gene clusters can be classified into two groups: (i) dispensable genes, where genes are shared by more than one strain but not all the strains; and (ii) strain specific genes. We discovered that 3059 (55.9 %) genes are dispensary genes while 50 (0.9 %) are strain-specific genes. Taking the 44 complete *L. monocytogenes* genomes used in this study as an example, we found 213 novel genes when second genome was added to the first genome, but the number of novel genes detected decreased to 33 when 43 genomes were added. The mathematical extrapolation illustrated in Fig. 4B yields the prediction of 33 novel genes that can be discovered for each additional genome added to the analysis, indicating that the open pan-genome of *L. monocytogenes* (all lineages combined) may be capable of continuously acquiring new genes.

### Comparative pathogenomic analysis

Some evidences suggest that the modern day pathogenic and non-pathogenic *Listeria* spp. have originated from a common pathogenic ancestor containing the key virulence genes which diverged long time ago [13]. It is also believed that the gene loss events including the loss of virulence associated genes such as the *prfA* gene cluster have played a critical role in the transition of *Listeria* species from facultative pathogen to saprotroph [13]. Interestingly, a number of non-pathogenic isolates still carry some of the virulence associated genes [13] and the genomic content of *L. monocytogenes* is closely related to some of the non-pathogenic species such as *L. innocua* and *L. marthii.* Here we wanted to have more comprehensive insights into the virulence profiles across *L. monocytogenes* strains and the non-pathogenic *Listeria* spp. using PathoProT of ListeriaBase. In this analysis, we used 44 *L. monocytogenes* strains (all have complete genome sequences) along with 3 strains of *L. innocua* and one strain of *L. marthii*. PathoProT heat map showed that the virulence profiles of the pathogenic and non-pathogenic strains were distinct and clearly segregated them into separate groups. A number of virulence factors were shared by all the selected strains. However, segregation the strains were based on the presence or absence of certain virulence factors.

**Fig. 5:**
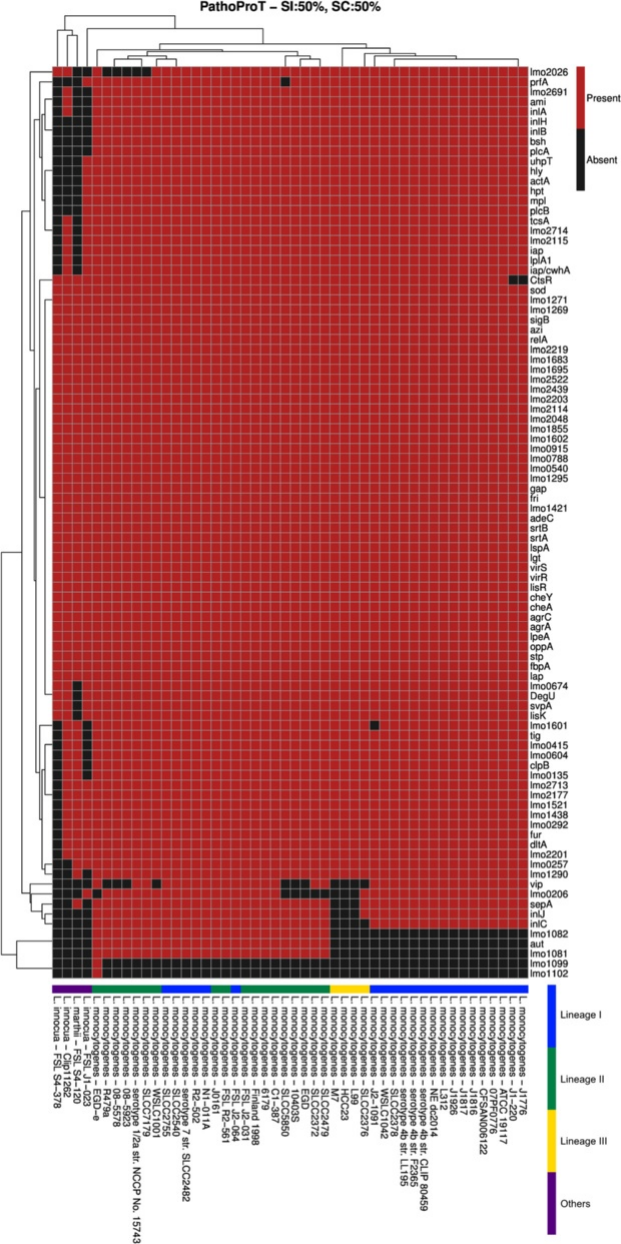
Virulence genes appear in different strains and clustered as heat map. There are a total of 92 virulence genes existing in *Listeria* species, and 78 of these virulence genes are conserved in all 44 *L. monocytogenes* strains. Lineages I and II of *L. monocytogenes* contain more virulence genes than lineages III, whereas majority of the virulence genes vital for pathogenicity are absent in *L. innocua* and *L. marthii*

The *L. innocua* and *L. marthii* strains in-spite of being non-pathogenic, due to their resemblance to *L. monocytogenes*, share a number of virulence genes with the pathogenic *L. monocytogenes* strains. Most of these shared virulence genes are related to regulation (*agrA, agrC, cheA, cheY, lisR, lisK, virR* and *virS*) and surface protein anchoring (*lgt, lspA, strA* and *srtB*) (Fig. 5). As anticipated, the *L. innocua* and *L. marthii* strains lack a number of important virulence factors that differentiate them from the pathogenic strains.

The *prfA* gene was conserved across all *L. monocytogenes* strains (except SLCC5850) and the non-pathogenic *L. innocua* FSL J1-023 (Fig. 5). It has previously been reported that *L. monocytogenes* can invade phagocytic and non-phagocytic cells in humans as well as ruminants where they self-replicate and spread directly from cell to cell, protecting themselves from host cell defense system during invasion. Each such infection process is regulated by the *prfA* gene, thereby regulating the expression of key virulence determinants of *L. monocytogenes* [62, 63]. However, *prfA* was absent in the lineage II SLCC5850. Previous studies have shown that the lack of *prfA* will attenuate the virulence of *L. monocytogenes* [14, 64]. Interestingly, lineage II SLCC5850 also lack the *vip* gene which is positively regulated by PrfA [65]. This gene was present in all strains of *L. monocytogenes* lineage I, but absent in the lineage III strains.

The *vip* gene was present in all lineage I strains and also most of the lineage II strains (Fig. 5). But all non-pathogenic *L. innocua* and *L. marthii* strains lack this gene. The *vip* gene encodes for an LPXTG surface protein Vip, which is anchored to the peptidoglycan layer of *Listeria* cell wall by sortase A and interacts with Gp96 of the host cell surface during invasion. Vip has also been reported to be involved in signaling events that may interfere with the host immune response in the course of the infection [65].

In addition, all *L. monocytogenes* strains had the virulence genes, *plcA* and *plcB*, but all non-pathogenic strains used in this analysis study showed the absence of the *plcA* gene. One of the non-pathogenic strains *L. innocua* FSL J1-023 did have the *plcB* gene, but the *plcA* gene was absent in the strain. Previous studies showed that *plcB* encodes for an exoenzyme which mediates dissolution of double-membrane secondary phagosomes [6, 66–68], whereas the *plcA* encodes for a phospholipase, which along with PlcB destabilizes the primary and the secondary phagosomes [6, 69, 70].

A virulence gene which is highly conserved across all pathogenic *L. monocytogenes* is the *bsh* gene. Interestingly, this gene was absent in all non-pathogenic strains of *L. innocua* and *L. marthii*. The *bsh* gene encodes for a bile salt hydrolase (BSH) that is important for the intestinal persistence of *L. monocytogenes* because of its involvement in resisting the acute toxicity of bile and bile salts [71, 72]. Besides the *bsh* gene, *ami* is another gene that was highly conserved in all *L. monocytogenes* strains. This gene encodes an autolytic amidase with an N-terminal catalytic domain and a C-terminal cell wall-anchoring domain made up GW modules and has been reported to be involved in the adhesion to eukaryotic cells via its cell wall-binding domain [73–75]. In the heat map, we observed that *L. monocytogenes* contain a large number of members of a protein family called internalins that are characterized by the presence of leucine-rich repeat domain distributed across the different lineages [76–81]. Of all the internalin proteins, InlA and InlB are well-studied and both exist in all three lineages (Fig. 5). InlA is a listerial surface protein required for invading non-phagocytic cells (e.g., epithelial cells), whereas InlB is necessary for invasion of *L. monocytogenes* to hepatocytes in the liver, fibroblasts and epithelioid cells [59, 60, 82–84]. Conversely, InlJ was identified as a new virulence factor among the internalin protein family [77]. InlJ was present in all lineage I and II strains and the lineage III SLCC2376. Although the function of InlJ is not fully understood, it has been reported to behave as an adhesin that helps bacteria to interact with host [85]. Interestingly, *inlA* was present in *L. innocua* Clip11262 but absent in the other *L. innocua* strains and also in *L. marthii*. But the *inlB* and *inlJ* were both absent in all *L. innocua* and *L. marthii* strains.

### *Aut* gene: a potential gene marker for differentiating *L. monocytogenes* serotype 4 with other serotypes

**Fig. 6:**
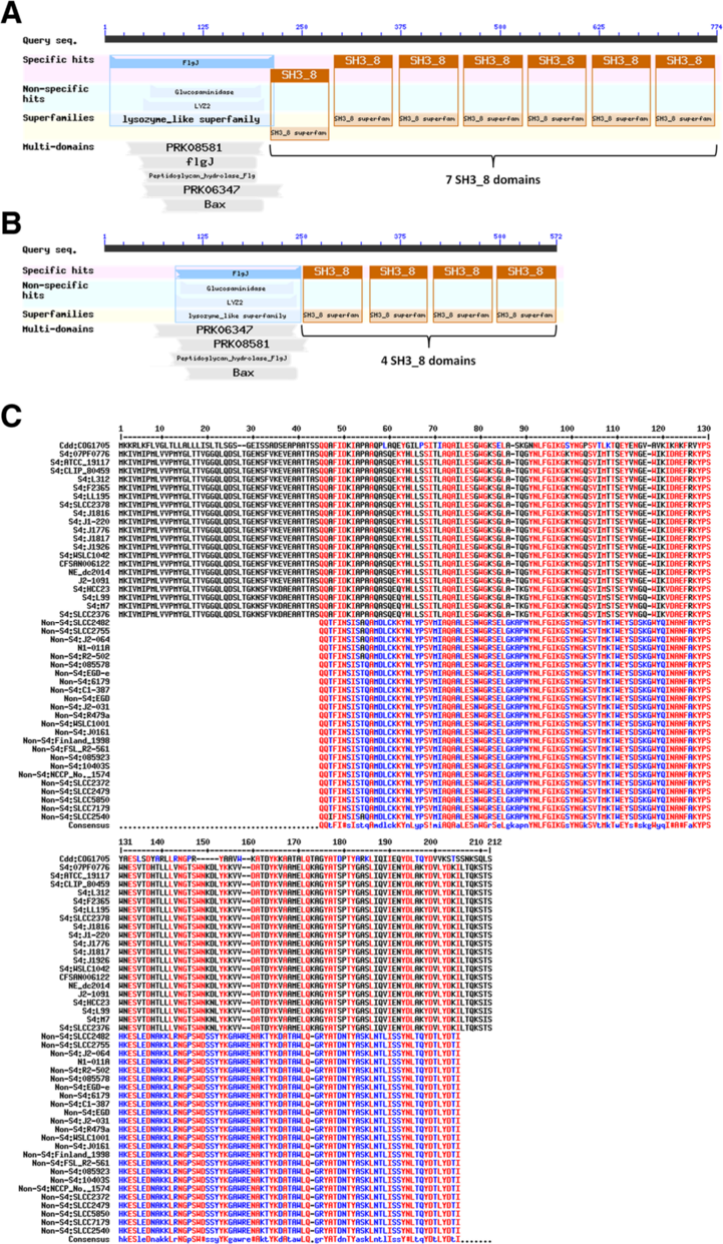
Difference between the Auto protein sequences of serotype 4 and non-serotype 4 strains of *L. monocytogenes*. **a** The domains present in the Auto protein sequence (FlgJ domain and 7 SH3_8 domains) in *L. monocytogenes* serotype 4 strains. **b** The domains present in the Auto protein sequence (FlgJ domain and 4 SH3_8 domains) in *L. monocytogenes* non-serotype 4 strains. **c** The multiple sequence alignment of the FlgJ domain of the Auto protein sequences of the serotype 4 and the non-serotype 4 strains of *L. monocytogenes* with FlgJ domain sequence COG1705 as the reference sequence

One interesting observation that emerged out of the comparative pathogenomics analysis was regarding the *aut* gene. The virulence gene *aut*, which encodes for the Auto protein, is crucial for the entry of *L. monocytogenes* to host cell, unaffected by the regulation of *prfA* gene [86]. The *aut* gene was absent in *L. innocua* and *L. marthii* strains. Interestingly, the *aut* gene was not detected by the PathoProT tool using the default parameters in the known serotype 4 strains of *L. monocytogenes* and also in some of the strains such as CFSAN006122, NE dc2014 and J2-1091 for which the serotypes are not clearly defined. However, we had noticed that the Auto proteins were present in the genome annotation files of those strains as predicted by the RAST server. The reason why the *aut* gene was not detected in those strains is because the levels of sequence identity and sequence completeness were below the default cut-off of PathoProT (50 % Sequence Identity and 50 % Sequence Completeness). We investigated further for the differences between the Auto protein sequences of the serotype 4 and the non-serotype 4 strains of *L. monocytogenes*. BLAST comparisons between the Auto protein sequences of the two groups of *L. monocytogenes* showed that they were not only homologs to each other, but also revealed unique sequence signatures that can differentiate the two groups. To better show the unique signatures in this paper, we generated the consensus sequences of the Auto protein for each group of *L. monocytogenes* (serotype 4 versus non-serotype 4) using the online tool MultAlin [87] and Conserved Domain Database of NCBI was used to search for the domains in the Auto protein sequences. In general, the Auto protein sequences of the members of the serotype 4 group were longer than the members of the non-serotype 4 group with both of the Auto protein structures containing FlgJ domain (essential for flagellar rod assembly), however the main difference was in the number of SH3_8 domains (7 SH3_8 domains in the known serotype 4 strains but only 4 SH3_8 domains in the known non-serotype 4 strains) (Fig. 6a, b). The length of the FlgJ domain in the Auto protein sequence of the non-serotype 4 strains was shorter than that of the serotype 4 strains (Fig. 6C). As for the strains CFSAN006122, NE dc2014 and J2-1091, the length of the FlgJ domain and the number of SH3_8 domains were found to be similar to that of the known serotype 4 strains. These observations strongly suggest that the Auto protein sequences of the serotype 4 and the non-serotype 4 strains of *L. monocytogenes* are distinctly different from each other especially due the differences in the number of the SH3_8 domains and may become a potential gene marker for differentiating serotype 4 strains from other serotypes.

## Conclusions

With the increasing number of *Listeria* genomes being sequenced, comparative genomics remains as a powerful approach for elucidating evolutionary mechanisms that shape the genomes. ListeriaBase aims to be one-stop genomic resource and analysis platform where useful genomic data and information can be obtained and analyzed using the provided analysis tools. We hope ListeriaBase will become a useful resource platform for the research communities and help to facilitate research on *Listeria*. ListeriaBase will be updated when new *Listeria* genomes become available. To further enhance ListeriaBase, suggestions on improving this database and requests for additional functions are certainly welcome. We hope that this effort will be able to provide a wide range of genome information in a central repository to accelerate future research on *Listeria* genomes.

## Availability and requirements

ListeriaBase can be accessed at http://listeria.um.edu.my. Users are free to download all the sequences and annotations used in this paper from the ListeriaBase website. ListeriaBase is best viewed by Mozilla Firefox® 10.x or higher, Safari 5.1 or higher, Chrome 18 or higher and any other equivalent browser software. If your browser is older, you may have trouble viewing many of our web site features properly. This web site is best viewed at a screen resolution of 1024 × 768 pixels or higher.

### Ethics

The present study did not involve any human subject, human material, or human data. No human patients or human samples were involved in the study. No animals or plants were also involved in the study. It also does not involve any new clinical tools or procedures.

## Additional files

Additional file 1: Table S1. Distribution of the 5 tRNA Island Clusters across the completely sequenced genomes of the three Lineages of *L. monocytogenes*. (PDF 207 kb) Additional file 2: Figure S1. tRNA Island Clusters. The 5 tRNA islands and their arrangements as identified in the *L. monocytogenes* strains. (TIFF 462 kb) Additional file 3: Figure S2. Phylogenetic tree based on 16S rRNA gene sequences of the representative strains of *Listeria* and strains of other genus (A) Phylogenetic tree constructed by using MEGA6. (B) Phylogenetic tree constructed by using ListeriaTree. (TIFF 1395 kb) Additional file 4: Figure S3. Phylogenetic trees of the 44 *Listeria monocytogenes* strains combining the 3 lineages using the ListeriaTree (A) Phylogenetic tree based on 16S rRNA gene (B) Phylogenetic tree based on *sigB* gene (C) Phylogenetic tree based on *gyrB* gene (D) Phylogenetic tree based on *actA* gene (E) Phylogenetic tree based on *groEL* gene. (TIFF 491 kb) Additional file 5: Figure S4. Pairwise genome comparison between the lineage III strains of *L. monocytogenes.* (A) PGC results *L. monocytogenes* SLCC2376 and *L. monocytogenes* L99. (B) PGC results *L. monocytogenes* SLCC2376 and *L. monocytogenes* M7. In both the figures (A) and (B) three noticeable gaps labelled as 1, 2 and 3 in circles can be observed. These three gaps in both the *L. monocytogenes* strains L99 and M7 were predicted to be prophages by PHAST; the first two being intact prophages while the third being questionable prophage. The green track indicates the histogram bars. Each 10 Kbp window in the diagram is assigned by a histogram bar. The height of each bar illustrates the total number of bases of the opposite genome aligned to this 10 Kbp window region. The upper border of the grey area delineates 10 Kbp height. If the height is higher than the 10 Kbp, it may indicate the genomic region is not specific or containing repetitive regions. A trough may indicate unmapped region which could be an insertion e.g. prophages. (TIFF 1623 kb) Additional file 6: Figure S5. Overview of the CDSs in the putative prophages predicted by PHAST, in the genome of *L. monocytogenes* HCC23. (TIFF 920 kb) Additional file 7: Table S2. Distribution of the Type II restriction modification system and CRISPR system across the completely sequenced genomes of the three lineages of *L. monocytogenes*. (PDF 231 kb)

## Abbreviations

AJAX: Asynchronous JavaScript and XML
BLAST: Basic local alignment search tool
bp: Base pair
CDS: Coding DNA sequence
CSS: Cascading style sheets
CRISPR-cas: Clustered regularly interspaced short palindromic repeat associated proteins
DNA: Deoxyribonucleic acid
HTML: HyperText markup language
Jbrowse: JavaScript-based genome browser
*L.*: *Listeria*
MVC: Model-view-controller
NCBI: National Center for Biotechnology Information
ORF: Open reading frame
PathoProT: Pathogenomics profiling tool
PGC: Pairwise genome comparison tool
PHP: HyperText preprocessor
RAST: Rapid annotation using subsystem technology
TI: tRNA genomic island
RNA: Ribonucleic acid
rRNA: Ribosomal ribonucleic acid
RM II: Type II restriction modification
spp.: Species
tRNA: Transfer ribonucleic acid
VFDB: Virulence factors database
MAFFT: Multiple alignment using fast fourier transform
