# Supplementary material for: Development of ListeriaBase and comparative analysis of Listeria monocytogenes
Source: BMC Genomics. 2015 Oct 6;16:755. doi: 10.1186/s12864-015-1959-5 (PMC4595109; doi:10.1186/s12864-015-1959-5)
Supplement: Additional file 1: Table S1. — Distribution of the 5 tRNA Island Clusters across the completely sequenced genomes of the three Lineages of L. monocytogenes. (PDF 207 kb) [file 12864_2015_1959_MOESM1_ESM.pdf]

Table S1. Distribution of the 5 tRNA Island Clusters across the completely sequenced genomes of the three Lineages of *L. monocytogenes*.

Lineage	Strains	tRNA Islands				
		1	2	3	4	5
**Lineage I**	07PF0776	√	√	√	√	√
	ATCC 19117	√	√	√	√	√
	CLIP 80459	√	√	√	√	√
	L312	√	√	√	√	√
	F2365	√	√	√	√	√
	LL195	√	√	√	√	√
	SLCC2482	√	√	√	√	√
	SLCC2378	√	√	√	√	√
	SLCC2540	√	√	√	√	√
	SLCC2755	√	√	√	√	√
	J1816	-	√	√	√	√
	J1-220	√	√	√	√	√
	CFSAN006122	√	√	√	√	√
	J2-064	-	√	√	√	√
	NE dc2014	√	√	√	√	√
	J2-1091	√	√	√	√	√
	J1776	√	√	√	√	√
	J1817	√	√	√	√	√
	J1926	√	√	√	√	√
	N1-011A	√	√	√	√	√
	R2-502	√	√	√	√	√
	WSLC1042	√	√	√	√	√
**Lineage II**	08-5578	-	√	√	√	√
	08-5923	-	√	√	√	√
	10403S	√	√	√	√	√
	EGD-e	√	√	√	√	√
	Finland 1998	√	√	√	√	√
	FSL R2-561	√	√	√	√	√
	J0161	-	√	√	√	√
	SLCC2372	√	√	√	√	√
	SLCC2479	√	√	√	√	√
	SLCC5850	√	√	√	√	√
	SLCC7179	√	√	√	√	√
	NCCP No. 15743	√	√	√	√	√
	6179	-	√	-	√	√
	C1-387	√	√	√	√	√
	EGD	√	√	√	√	√
	J2-031	√	√	√	√	√
	R479a	-	√	√	√	√
	WSLC1001	√	√	√	√	√
**Lineage III**	HCC23	√	√	√	√	√
	L99	√	√	√	√	√
	M7	√	√	√	√	√
	SLCC2376	√	√	√	√	√
