# Supplementary figures and images for: Development of ListeriaBase and comparative analysis of Listeria monocytogenes
Source: BMC Genomics. 2015 Oct 6;16:755. doi: 10.1186/s12864-015-1959-5 (PMC4595109; doi:10.1186/s12864-015-1959-5)

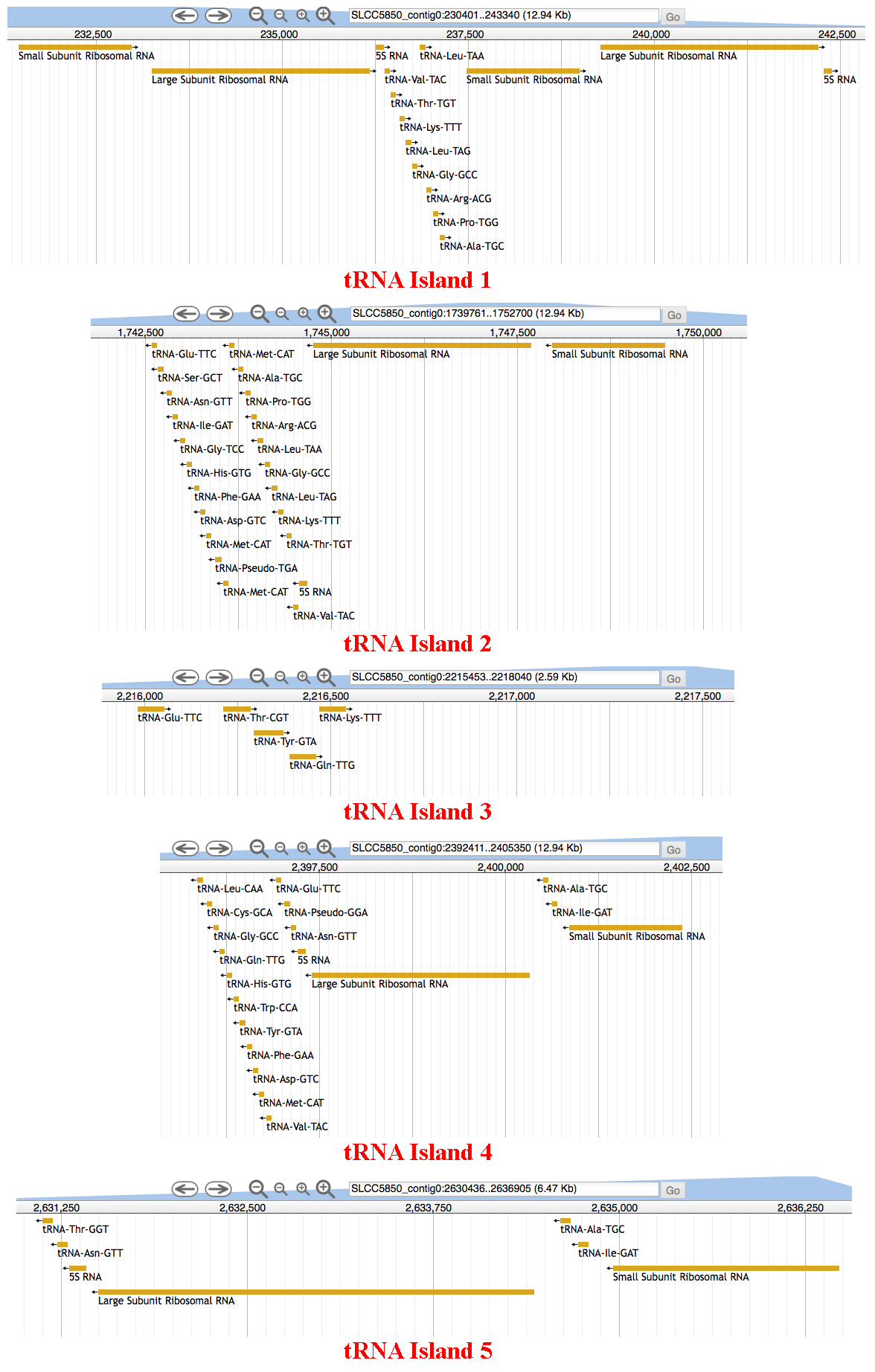

Supplement: Additional file 2: Figure S1. — tRNA Island Clusters. The 5 tRNA islands and their arrangements as identified in the L. monocytogenes strains. (TIFF 462 kb) [file 12864_2015_1959_MOESM2_ESM.tif]

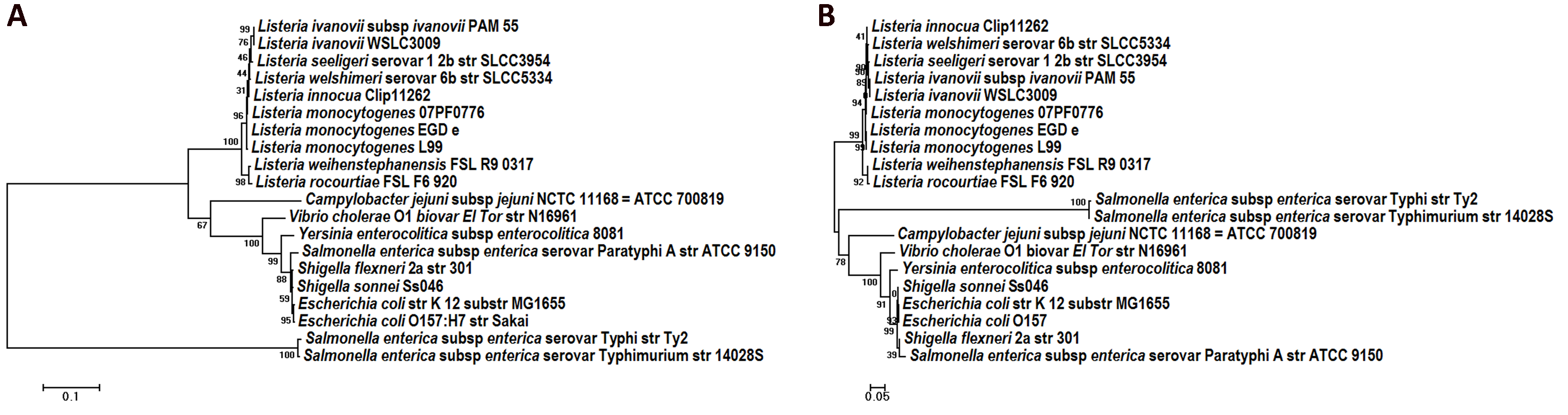

Supplement: Additional file 3: Figure S2. — Phylogenetic tree based on 16S rRNA gene sequences of the representative strains of Listeria and strains of other genus (A) Phylogenetic tree constructed by using MEGA6. (B) Phylogenetic tree constructed by using ListeriaTree. (TIFF 1395 kb) [file 12864_2015_1959_MOESM3_ESM.tif]

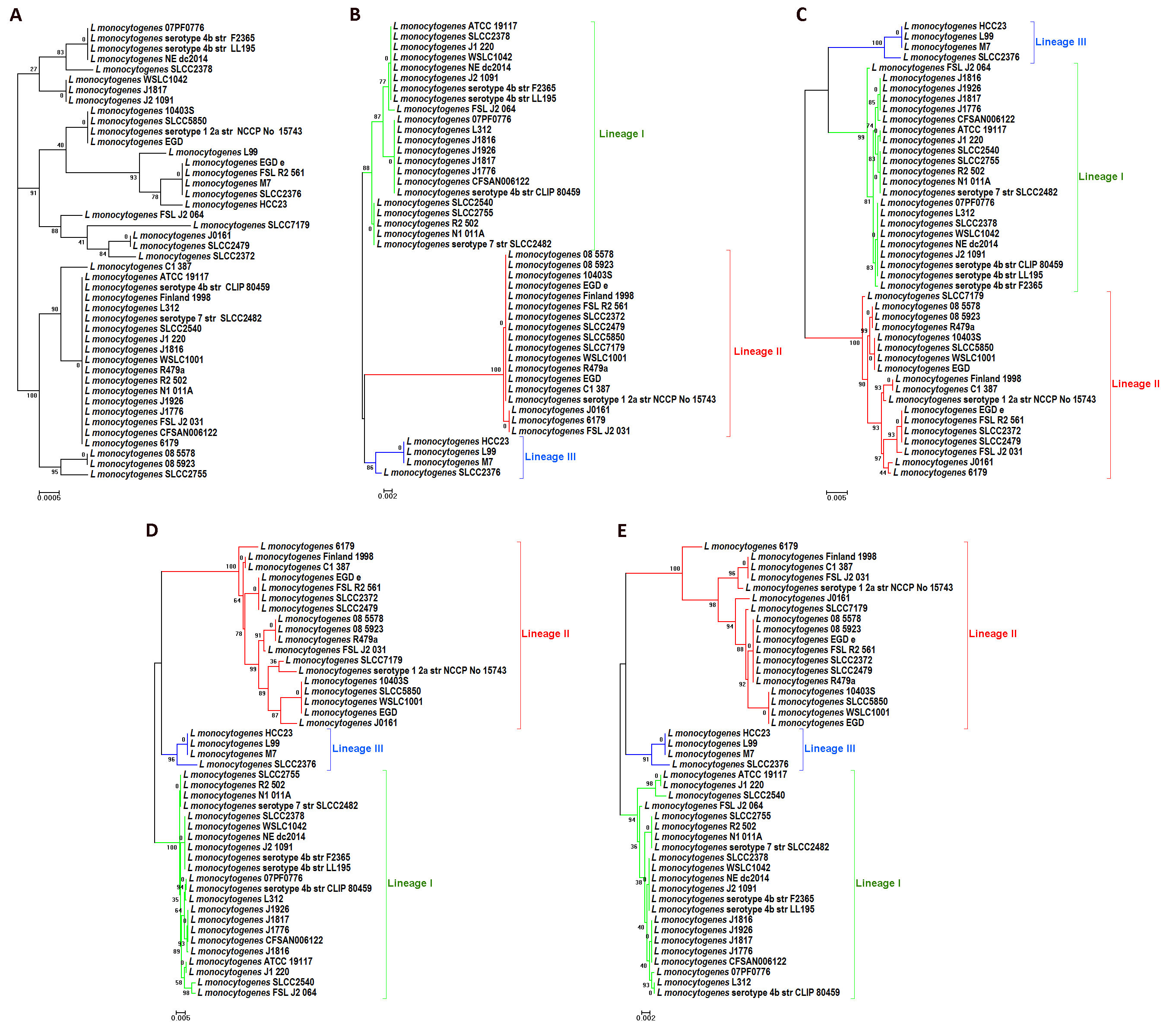

Supplement: Additional file 4: Figure S3. — Phylogenetic trees of the 44 Listeria monocytogenes strains combining the 3 lineages using the ListeriaTree (A) Phylogenetic tree based on 16S rRNA gene (B) Phylogenetic tree based on sigB gene (C) Phylogenetic tree based on gyrB gene (D) Phylogenetic tree based on actA gene (E) Phylogenetic tree based on groEL gene. (TIFF 491 kb) [file 12864_2015_1959_MOESM4_ESM.tif]

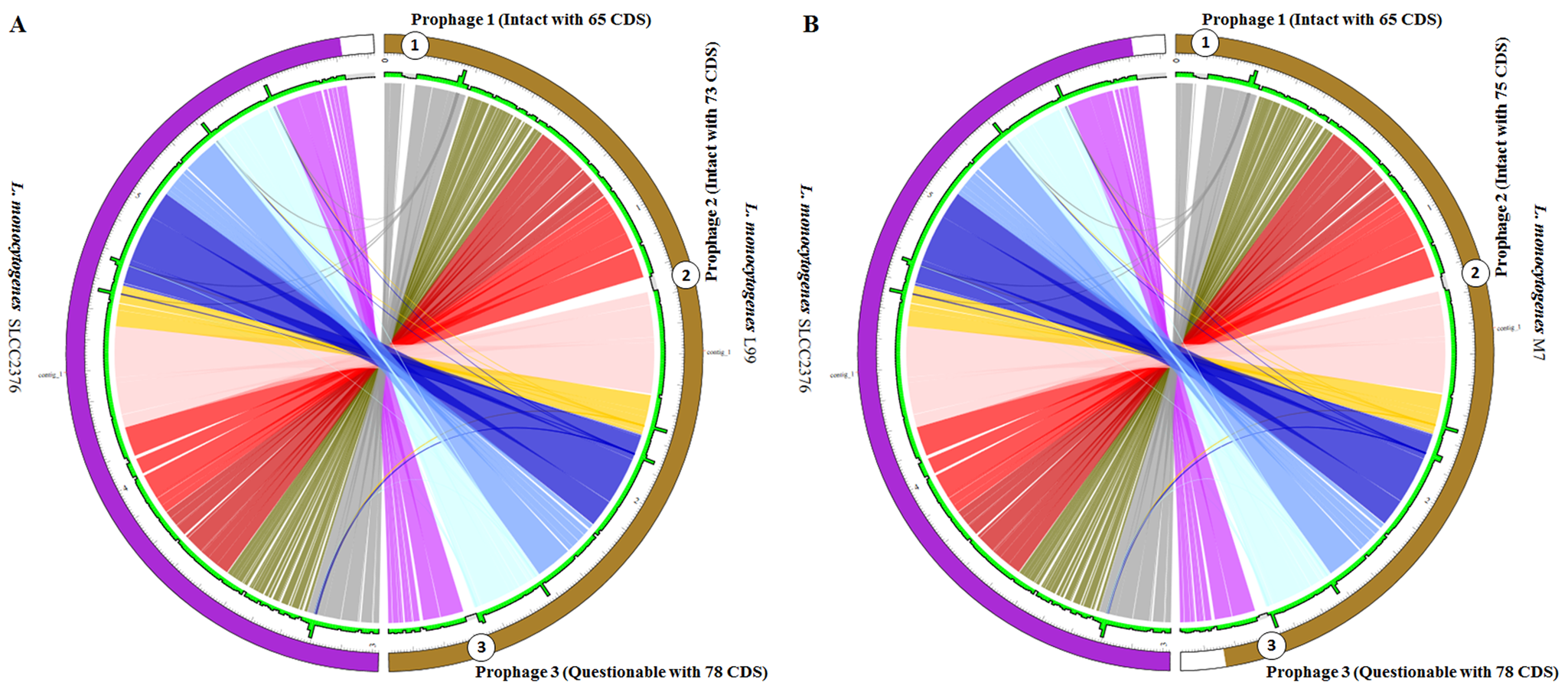

Supplement: Additional file 5: Figure S4. — Pairwise genome comparison between the lineage III strains of L. monocytogenes. (A) PGC results L. monocytogenes SLCC2376 and L. monocytogenes L99. (B) PGC results L. monocytogenes SLCC2376 and L. monocytogenes M7. In both the figures (A) and (B) three noticeable gaps labelled as 1, 2 and 3 in circles can be observed. These three gaps in both the L. monocytogenes strains L99 and M7 were predicted to be prophages by PHAST; the first two being intact prophages while the third being questionable prophage. The green track indicates the histogram bars. Each 10 Kbp window in the diagram is assigned by a histogram bar. The height of each bar illustrates the total number of bases of the opposite genome aligned to this 10 Kbp window region. The upper border of the grey area delineates 10 Kbp height. If the height is higher than the 10 Kbp, it may indicate the genomic region is not specific or containing repetitive regions. A trough may indicate unmapped region which could be an insertion e.g. prophages. (TIFF 1623 kb) [file 12864_2015_1959_MOESM5_ESM.tif]

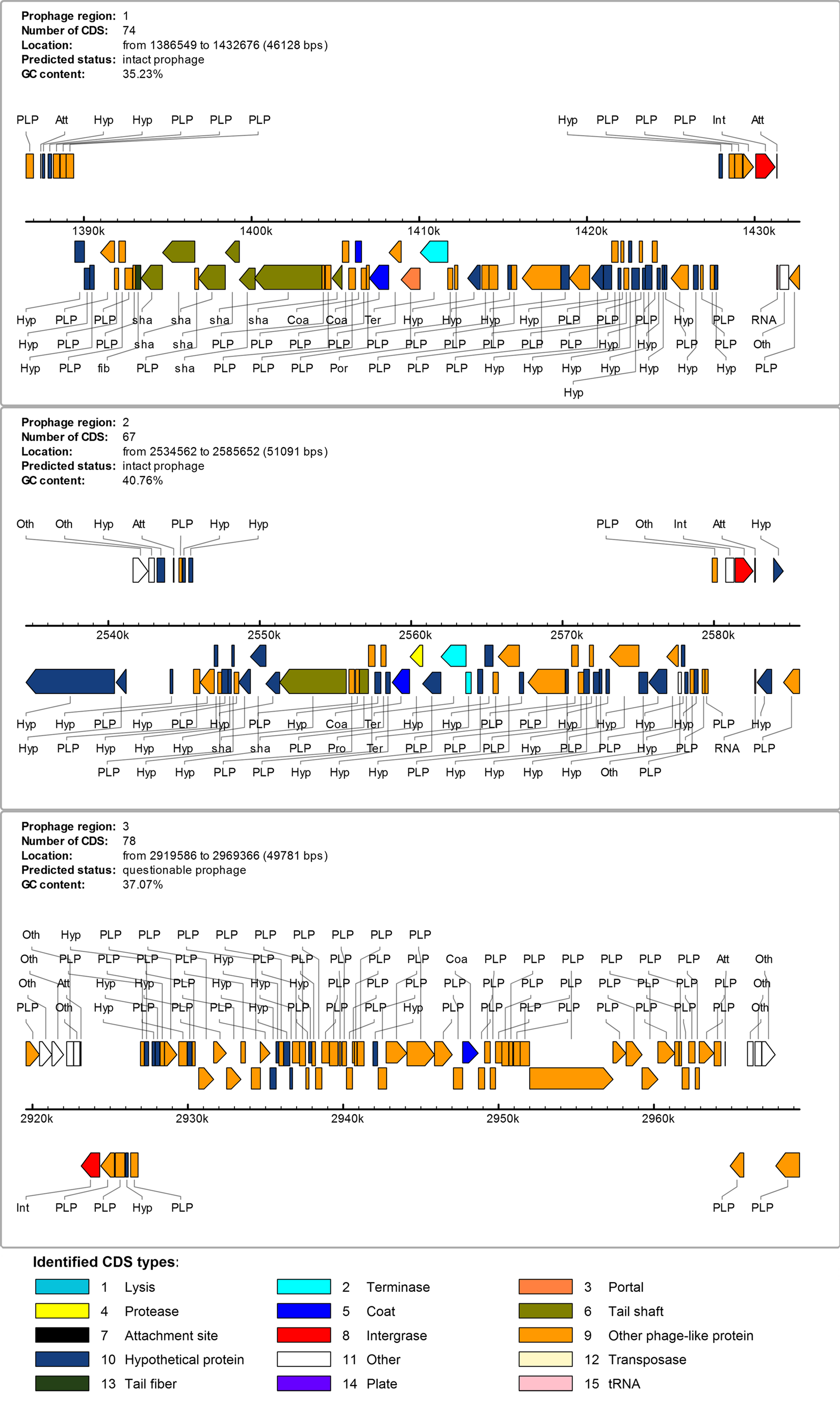

Supplement: Additional file 6: Figure S5. — Overview of the CDSs in the putative prophages predicted by PHAST, in the genome of L. monocytogenes HCC23. (TIFF 920 kb) [file 12864_2015_1959_MOESM6_ESM.tif]
