# Supplementary material for: Development of ListeriaBase and comparative analysis of Listeria monocytogenes
Source: BMC Genomics. 2015 Oct 6;16:755. doi: 10.1186/s12864-015-1959-5 (PMC4595109; doi:10.1186/s12864-015-1959-5)
Supplement: Additional file 7: Table S2. — Distribution of the Type II restriction modification system and CRISPR system across the completely sequenced genomes of the three lineages of L. monocytogenes. (PDF 231 kb) [file 12864_2015_1959_MOESM7_ESM.pdf]

**Table S2. Distribution of the Type II restriction modification system and CRISPR system across the completely sequenced genomes of the three Lineages of *L. monocytogenes*.**

| Lineage | Strains | Type II restriction modification system | | CRISPR system | | | | | |
|---|---|---|---|---|---|---|---|---|---|
| | | Type II restriction enzyme NgoPII (EC 3.1.21.4) | DNA-cytosine methyltransferase (EC 2.1.1.37) | CRISPR repeat RNA endoribonuclease Cas6 | CRISPR-associated negative autoregulator | CRISPR-associated protein MTH1087 | CRISPR-associated helicase Cas3 | CRISPR-associated protein Cas1 | CRISPR-associated protein Cas2 |
| **Lineage I** | 07PF0776 | - | √ | - | - | - | - | - | - |
| | ATCC 19117 | - | √ | - | - | - | - | - | - |
| | CLIP 80459 | - | √ | - | - | - | - | - | - |
| | L312 | - | √ | - | - | - | - | - | - |
| | F2365 | - | √ | - | - | - | - | - | - |
| | LL195 | - | √ | - | - | - | - | - | - |
| | SLCC2482 | - | - | √ | √ | √ | √ | √ | √ |
| | SLCC2378 | - | √ | - | - | - | - | - | - |
| | SLCC2540 | - | - | - | - | - | - | √ | √ |
| | SLCC2755 | - | - | √ | √ | √ | √ | √ | √ |
| | J1816 | - | - | - | - | - | - | - | - |
| | J1-220 | - | √ | - | - | - | - | - | - |
| | CFSAN006122 | - | - | - | - | - | - | - | - |
| | J2-064 | - | - | - | - | - | - | - | - |
| | NE dc2014 | - | √ | - | - | - | - | - | - |
| | J2-1091 | - | √ | - | - | - | - | - | - |
| | J1776 | - | - | - | - | - | - | - | - |
| | J1817 | - | - | - | - | - | - | - | - |
| | J1926 | - | - | - | - | - | - | - | - |
| | N1-011A | - | - | √ | √ | √ | √ | √ | √ |
| | R2-502 | - | - | √ | √ | √ | √ | √ | √ |
| | WSLC1042 | - | √ | - | - | - | - | - | - |
| **Lineage II** | 08-5578 | - | - | - | - | - | - | - | - |
| | 08-5923 | - | - | - | - | - | - | - | - |
| | 10403S | - | - | - | - | - | - | √ | √ |
| | EGD-e | - | - | - | - | - | - | - | - |
| | Finland 1998 | - | - | √ | √ | √ | √ | √ | √ |
| | FSL R2-561 | - | - | - | - | - | - | - | - |
| | J0161 | - | - | √ | √ | √ | √ | √ | √ |
| | SLCC2372 | - | - | - | - | - | - | - | - |
| | SLCC2479 | - | - | - | - | - | - | - | - |
| | SLCC5850 | - | - | - | - | - | - | √ | √ |
| | SLCC7179 | - | √ | - | - | - | - | - | - |
| | NCCP No. 15743 | - | - | - | - | - | - | - | - |
| | 6179 | - | √ | √ | √ | √ | √ | √ | √ |
| | C1-387 | - | - | √ | √ | √ | √ | √ | √ |
| | EGD | - | - | - | - | - | - | √ | √ |
| | J2-031 | - | - | - | - | - | - | √ | - |
| | R479a | - | - | - | - | - | - | - | - |
| | WSLC1001 | - | - | - | - | - | - | √ | √ |
| **Lineage III** | HCC23 | √ | √ | √ | √ | √ | √ | √ | √ |
| | L99 | √ | √ | √ | √ | √ | √ | √ | √ |
| | M7 | √ | √ | √ | √ | √ | √ | √ | √ |
| | SLCC2376 | √ | √ | - | - | - | - | - | - |
